# Correction: Solution of time-fractional gas dynamics equation using Elzaki decomposition method with Caputo-Fabrizio fractional derivative

**DOI:** 10.1371/journal.pone.0321042

**Published:** 2025-03-25

**Authors:** Maasoomah Sadaf, Zahida Perveen, Ghazala Akram, Ume Habiba, Muhammad Abbas, Homan Emadifar

An additional affiliation is missing for the sixth author. Homan Emadifar is also affiliated with the Department of Mathematics, Saveetha School of Engineering, Saveetha Institute of Medical and Technical Sciences, Saveetha University, Chennai, 602 105, Tamil Nadu, India.
