# Supplementary material for: Solution of time-fractional gas dynamics equation using Elzaki decomposition method with Caputo-Fabrizio fractional derivative
Source: PLoS One. 2024 May 30;19(5):e0300436. doi: 10.1371/journal.pone.0300436 (PMC11139311; doi:10.1371/journal.pone.0300436)
Supplement: Attachment — Submitted filename: Response_to_reviewers (1).pdf [file pone.0300436.s001.pdf]

Response to reviewers' comments on "Solution of time-fractional gas dynamics equation using Elzaki decomposition method with Caputo-Fabrizio fractional derivative"

Dear Editor

Hope you are doing well. Thank you for your email. The authors are grateful to the reviewers for their valuable comments and suggestions. The manuscript is revised in the light of reviewers' comments. All the suggestions have been incorporated in the revised manuscript. The detailed response to reviewers' comments is given below.

Thanks and Regards

Response to Reviewer 1

1_Add mathematical proofs or citations where appropriate to improve the rigor.

Response: First of all, thank you for your careful and professional review which has greatly helped us to improve the quality of this manuscript. The relevant mathematical definitions and results are added in Section 2 of the revised manuscript with suitable citations. Moreover, the relevant citations are added throughout the manuscript at appropriate places.

2- Include some theoretical analysis of the accuracy and convergence of the approximation technique.
Response: The accuracy of the approximation technique is established by the comparison of the obtained results with the results available in literature as described in the newly added Section 5: "Discussion of the results". Moreover, the newly proposed method requires more time and effort to develop a convergence theorem which will be included in our future work.

3- Demonstrate the method on a wider variety of test problems involving different types of nonlinearities, boundary conditions etc.

Response: The reason for taking the presented test problems is explained Section 5 of the revised manuscript as follows: "These two examples have been previously studied with different fractional time-derivatives and their exact solutions are known at $\beta = 1$ [9, 13, 17]. Thus, the selected numerical examples allow a comparison of the obtained results with the results available in literature."
Since the comparison confirms the accuracy and reliability of the proposed technique to solve the gas-dynamics equation, other test problems can be dealt with in a similar fashion.

4- Provide physical explanations of the effect observed when changing the fractional order β.

Response: Since the theory of fractional calculus and fractional order models is still evolving, the physical applications of the use of fractional derivative are yet to be fully explored. However, we can observe that the solution surface for the gas-dynamics equation continuously changes for increasing value of fractional order β. Ultimately, the solution surface at $\beta = 1$ coincides with the solution of the classical integer order gas-dynamics equation which confirms that CF derivative is indeed a generalization of the classical integer order derivative.
The same explanation is also added in Section 5 of the revised manuscript.

5- Expand the introduction to provide a more thorough literature review of existing methods for solving fractional gas dynamics equations.

Response: The literature review for the fractional gas-dynamics equation is improved in the introduction of the revised manuscript.

6- Carefully proofread the article to fix typos, formatting, and grammar issues.

Response: We have tried to correct all typos, formatting, and grammar mistakes in the revised manuscript.

7- Overall, the paper presents a novel application of a useful analytical technique but needs some more mathematical depth and discussion of results to improve its potential for publication. Addressing the above points would strengthen the paper.

Response: The authors are grateful to you for your valuable comments and suggestions. We have tried our best to suitably address all the comments and suggestions. We hope that the revised manuscript will meet your approval.

Thank you very much for your valuable comments and suggestions

Response to Reviewer 2

I would recommend publication of the manuscript in PLOS ONE provided that the authors can address the following issues properly.

Response: Thank you for your careful and professional review which has greatly helped us to improve the quality of this manuscript. We hope that the revised manuscript will meet your approval.

1. Please check the Abstract and Conclusion in terms of the grammar English language for example the abstract change to (In this article, Elzaki decomposition method (EDM) has been applied to approximate the analytical solution of the time-fractional gas-dynamics equation. The time-fractional derivative is used in the Caputo-Fabrizio sense. The proposed method is implemented on homogenous and non-homogenous cases of the time-fractional gas-dynamics equation. A comparison between the exact and approximate solutions is also provided to show the validity and accuracy of the technique. A graphical representation of all the retrieved solutions is shown for different values of the fractional parameter. The time development of all solutions is also represented in 2D graphs. The obtained results may help understand the physical systems governed by the gas-dynamics equation.)

Response: Thank you for your careful review. The abstract is corrected in the revised manuscript according to the suggestion. The conclusion is also revised.

2. the results mentioned in this manuscript are confirmed with the review.

Response: The accuracy of the obtained results is confirmed by comparison with the results already available in literature in Section 5 of the revised manuscript.

3. To complete the literature review, the following papers should be added to the references list:
• Communications in Theoretical Physics 74 (7), 075003.
• Advances in Difference Equations 331 (doi.org/10.1186/s13662-020-02789-5).

Response: The suggested relevant papers are added in the introduction and references to improve the literature review of the manuscript.

Thank you very much for your valuable comments and suggestions
